# Detection of tetrabromobisphenol A and its mono- and dimethyl derivatives in fish, sediment and suspended particulate matter from European freshwaters and estuaries

**DOI:** 10.1007/s00216-017-0312-z

**Published:** 2017-03-21

**Authors:** Matthias Kotthoff, Heinz Rüdel, Heinrich Jürling

**Affiliations:** Fraunhofer Institute for Molecular Biology and Applied Ecology IME, Auf dem Aberg 1, 57392 Schmallenberg, Germany

**Keywords:** Brominated flame retardants, Tetrabromobisphenol A, Environmental monitoring, Atmospheric pressure photoionization, High-resolution mass spectrometry

## Abstract

An analytical method was developed for the determination of tetrabromobisphenol A (TBBPA), 3,3′,5,5′-tetrabromobisphenol-A-monomethyl ether (MM-TBBPA) and 3,3′,5,5′-tetrabromobisphenol-A-dimethyl ether (DM-TBBPA), and its valid application on fish muscle matrix (bream and sole), suspended particulate matter (SPM) and surface sediment layer samples, using only 0.5 g sample material, is demonstrated. Here, for the first time, DM-TBBPA could be determined by an LC-MS/MS-based method applying atmospheric pressure photoionization (APPI), using the same sample extracts for all three analytes. Samplings covered freshwater fish (bream; annually, period 2007–2013) and SPM or sediment (every second year in the period 2008–2014) at selected European sites (rivers: Tees/UK, Mersey/UK, Western Scheldt/NL, Götaälv/SE, Rhône/FR; Lake Belau/DE). TBBPA could be quantified in 13 of 36 bream samples (range about 0.5–1.2 μg kg^−1^ ww) and 7 of 7 sole muscle samples (range about 0.5–0.7 μg kg^−1^ ww). Further, it could be quantified in 11 of the 14 SPM samples (range about 0.5–9.4 μg kg^−1^ dw) and in both of the surface sediment layer samples (2.3–2.6 μg kg^−1^ dw). MM-TBBPA could be quantified in 12 of 36 bream and 4 of 7 sole muscle samples (range about 0.8–1.8 μg kg^−1^ ww). Further, it could be quantified in 10 of the 14 river SPM samples (range about 2.3–4.5 μg kg^−1^ dw) and in both lake surface sediment layer samples (5.2–5.5 μg kg^−1^ dw). DM-TBBPA was rarely detectable and could not be quantified above the limit of quantification in any sample.

**Electronic supplementary material:**

The online version of this article (doi:10.1007/s00216-017-0312-z) contains supplementary material, which is available to authorized users.

## Introduction

Tetrabromobisphenol A (TBBPA, IUPAC: 2,2′,6,6′-tetrabromo-4,4′-isopropylidenediphenol, CAS: 79-94-7) is one of the most widely used brominated flame retardants (BFRs) with an annual usage of about 130,000 to 170,000 t in 2004/2005 [1, 2]. TBBPA has been widely applied as a flame retardant in microelectronic and electric applications. TBBPA has been found not only in many environmental matrices in low concentrations, such as water, soil, air, sediments, or marine and terrestrial biota, but also in human breast milk [3–8].

TBBPA in particular is mainly used as a reactive ingredient in microelectronic applications such as printed circuit boards (epoxy, polycarbonate and phenolic resins) and in rare cases as additive BFR, e.g. in the production of acrylonitrile-butadiene-styrene (ABS) resins [9, 10]. Depending on the application, concentrations of TBBPA within the polymers may rise up to 20% by weight [2]. TBBPA meets the persistence criteria but is not classified as bioaccumulative or toxic in the EU [11]. However, the environmental risk assessment report of the EU states that TBBPA is very toxic to aquatic organisms and may cause long-term adverse effects to the aquatic environment [12]. Furthermore, possible risk for surface water, sediment and soil was concluded in the EU risk assessment. For surface water and sediment, this conclusion applies to compounding sites where TBBPA is used as an additive flame retardant in ABS plastics. For soil compartment, this conclusion applies to the use of TBBPA as an additive flame retardant in ABS from compounding and conversion sites. The conclusion for conversion sites is dependent on whether or not sewage sludge from the site is applied to agricultural land [13]. The major producers have since followed up with the downstream users in a voluntary emission reduction programme focused on monitoring the entries in these environmental compartments at the respective customer sites [14].

Once TBBPA reaches the environment, different degradation or metabolic routes may account. The major route of degradation is the reductive dehalogenation in sewage sludge or sediments, finally leading to bisphenol A (BPA) [15, 16]. TBBPA can be O-methylated to form TBBPA-monomethyl ether (MM-TBBPA) and TBBPA-dimethyl ether (DM-TBBPA), very much depending on the specific conditions [17, 18]. In silico screening suggests that due to their higher lipophilicity, the metabolism to MM- and DM-TBBPA may lead to molecules with higher persistence that may bioaccumulate so the metabolites might meet the criteria for PBT substances [12].

Some environmental monitoring data exist for TBBPA, but comprehensive data are scarce or very scarce for DM- and MM-TBBPA in environmental matrices [2]. TBBPA has been found in numerous matrices and locations. Two very detailed reviews on TBBPA have recently been published and provide a comprehensive overview on the distribution, fate and release [13], as well as the occurrence, potential human exposure and especially the analysis, of TBBPA [10]. One of the few available published studies on DM-TBBPA found low levels in falcon eggs from Greenland [19].

Most analytical methods for TBBPA apply liquid chromatographic (LC) separation coupled to mass spectrometric (MS) or optical detection techniques subsequent to elaborate sample preparation in all researched matrices, rarely GC-MS after derivatization, e.g. with diazomethane or *N*-methyl-*N*-(trimethylsilyl) trifluoroacetamide [2, 20, 21]. Methods for TBBPA are published for many matrices and reach satisfactory limits of quantification (LOQs), e.g. 0.06 μg kg^−1^ wet weight (ww) in fish samples, by applying a QuEChERS/LLE sample preparation and measurement via LC-MS/MS [22]. However, up to date, the sensitivities of LC-MS-based analytical methods were insufficient for MM- and DM-TBBPA to be used as methods of choice. So, MM- and DM-TBBPA have so far been analysed via gas chromatographic (GC) separation coupled to MS-detection techniques [19, 23, 24]. In those studies, either separate sample preparation methods were applied with subsequent derivatization of the subspecies to adopt their chemical properties for GC or simultaneous sample preparation with differential instrumental determination was applied.

In this retrospective monitoring study, fish—bream and sole—as well as sediment and suspended particulate matter (SPM) were analysed for TBBPA and its metabolites MM- and DM-TBBPA in a multi-target approach to provide a comprehensive overview of the three dependent substances in the European aquatic environment. The samples reflect time series of different European estuaries and one lake and span a sampling period from 2007 to 2013. Two goals are targeted with this study: first, we provide a common method for the determination of a related analyte spectrum, and second, the method is applied on a series of different time points and European sampling sites to allow a brief insight into the environmental occurrence of TBBPA and its metabolites.

## Materials and methods

### Sampling

Sampling of bream (*Abramis brama*) was performed between 2007 and 2013 at six European sites, i.e. in the river estuary sites Scheldt (The Netherlands: 51° 23′ N, 4° 14′ E), Rhône (France: 43° 38′ N, 4° 36′ E), Göta älv (Sweden, no sampling 2009–2011; 57° 43′ N, 11° 59′ E), Tees (UK: 54° 33′ N, 1° 18′ E) and Mersey (UK, no sampling 2009–2011; 53° 23′ N, 2° 34′ E), and in the freshwater Lake Belau (Germany: 54° 06′ N, 10° 15′ E). At the Scheldt site, also soles (*Solea solea*) were caught between 2007 and 2013. The sampling procedure for fish, including detailed description of sampling sites and raw sample homogenization, is described by Rudel et al. [25].

If available, 15 fish per site were collected after the spawning season. The filets were dissected and combined for the preparation of annual pool samples. Samples were stored at temperatures <−150 °C. Based on stable isotope data for nitrogen, bream had a trophic level (TL) of about 2.5–3.9 at the investigated sites [26]. The generic TL according to FishBase based on diet studies is 3.1 ± 0.1 [27].

From the same river sampling sites, also SPM was sampled, and sediment for analysis was taken from the sampling site Lake Belau. The SPM sampling followed the procedure described by Schulze et al. [28]. At the river sites, SPM sampling campaigns were performed covering periods of 1 year each (from autumn to autumn, respectively; the sampling campaigns are designated by the year in which the major part of the sampling occurred) using stainless steel traps which were emptied every 3 months. SPM was kept frozen at <−150 °C after sampling. After freeze-drying, SPM samples were prepared routinely as annual composite samples from equal amounts of the four 3-month periods. At Lake Belau, surface sediment was collected by core sampling (every second year in autumn/winter) and frozen at <−150 °C directly after sampling. The upper sediment layer of about 2 cm was cut with a stone saw from the frozen core, freeze-dried and homogenized. For each sampling, 16 cores with an inner diameter of 4.5 cm were collected. Samples were stored at temperatures <−150 °C and analysed within 6 months after sampling. The sediment samples are designated by the year in which the major part of the sedimentation occurred. Two Lake Belau surface sediment layers were investigated. Previous investigations within the German Environmental Specimen Bank programme at Lake Belau revealed that the upper sediment layer of 1–2 cm corresponds to the freshly deposited material in a 12-month period. The upper sediment layer of about 2 cm was therefore separated in the laboratory by cutting under cryogenic conditions. Sixteen frozen cores were processed. Then, the top layers of four cores each were combined, homogenized and finally freeze-dried.

### Laboratory sample preparation for analysis

Reference materials used are TBBPA (Sigma Aldrich, Saint Louis, MA; #11223, Lot BCBJ4254V, purity 98.8%), MM-TBBPA (provided from BSEF (Chemtura); #146823-76-9, 98.0%) and DM-TBBPA (provided from BSEF (Chemtura); #146823-76-9, 97.9%). A low amount of starting material was required to carefully handle the valuable samples. Approximately 0.5 g of sample material (bream or sole muscle, SPM or sediment) was transferred into a 15-mL polypropylene (PP) centrifugation tube. Only for fish muscle samples: 500 μL of concentrated sulphuric acid was added and the samples treated for 10 min in an ultrasonic bath (in order to degrade lipid materials). Then, 100 μL of an internal standard solution (^13^C-labelled TBBPA, Cambridge Isotope Laboratories, Inc., Andover, MA; 50 μg mL^−1^ with a specific purity of ≥98%; *c* = 100 μg L^−1^) and 5 mL extraction solvent (dichloromethane/*n*-hexane, 3 + 1, *v*/*v*) were added. The samples were homogenized with an Ultra Turrax device for 1 min and thoroughly shaken for 20 min. The resulting homogenate was centrifuged at 4000 rpm for 5 min. The supernatants were transferred into another 15-mL PP centrifugation tube and the extraction procedure was repeated twice. Fish matrix was worked up in acidic conditions to lyse the fish tissue. This also adds to keep TBBPA protonated. However, an adjustment of pH was not required in other matrices.

The combined ca. 15 mL extraction solvents were evaporated in a nitrogen evaporator at 40 °C to dryness and the residues were resolved in 1 mL acetonitrile-water (50 + 50, *v*/*v*) using an ultrasonic bath for 5 min. Each extract was filtered using 25 mm × 0.45 μm regenerated cellulose (RC) filter cartridges directly into 1.5-mL autosampler vials for analysis via LC-HR-MS or LC-APPI-MS/MS analysis for TBBPA and MM-TBBPA or DM-TBBPA, respectively. All sample extracts were measured in duplicate and means were reported.

### Instrumental analysis of TBBPA and MM-TBBPA

Analysis of TBBPA and MM-TBBPA was performed by LC-HR-MS/MS on an Orbitrap Q-Exactive instrument (Thermo Fisher Scientific Inc.) connected with an UPLC Acquity chromatographic device (Waters). Chromatographic conditions are as follows. Column: 100 × 2.1 mm BEH C18, 1.7 μm (Waters); flow 0.35 mL min^−1^; injection volume 10 μL; column temperature 55 °C; and ionization mode: electrospray negative (ESI^−^). Solvent gradient: 0 to 10 min 100% A [water/methanol (95 + 5, *v*/*v*) + 2 mM ammonium acetate], 10 to 12 min ramp to 100% B (methanol + 2 mM ammonium acetate) 12 to 15 min 100% B, then 100% A.

Definitions and accurate masses of analytes used were as follows: TBBPA (C_15_H_12_O_2_Br_4_; 538.74957 Da) and for quantification their respective bromine-81 isotopes (^81^Br) (C_15_H_11_O_2_Br_2_^81^Br_2_; 542.74554 Da), MM-TBBPA (C_16_H_14_O_2_Br_4_; 552.76430 Da), ^81^Br-MM-TBBPA (C_16_H_13_O_2_Br_2_^81^Br_2_Br_2_; 556.76030 Da), as internal standard ^13^C-TBBPA (C_3_^13^C_12_H_12_O_2_Br_4_; 550.78896 Da), and ^13^C^81^Br-TBBPA (C_3_^13^C_12_H_12_O_2_Br_2_^81^Br_2_Br_2_; 554.78540 Da).

A basic calibration with TBBPA and MM-TBBPA was performed in the concentration range from 0.1 to 10.0 μg L^−1^. Each calibration solution consisted of 10 calibration points and contained 10 μg L^−1^ internal standard (^13^C-TBBPA).

### Instrumental analysis of DM-TBBPA

Analysis of DM-TBBPA was performed by LC-APPI-MS/MS on a Waters TQ-S instrument (Waters) connected with an UPLC Acquity chromatographic device (Waters). Chromatographic conditions are as follows. Column: 50 × 2.1 mm BEH C18, 1.7 μm (Waters); flow 0.05 mL min^−1^; injection volume 25 μL; column temperature 55 °C; ionization mode: atmospheric pressure photoionization positive (APPI+) using an APPI/APCI dual source (Waters). Solvent gradient: 0 to 10 min 100% A, 10 to 17 min ramp to 100% B 17 to 30 min 100% B, then 100% A.

Definitions and mass transitions of analytes used were as follows: for quantification DM-TBBPA (C_17_H_16_O_2_Br_4_, *m*/*z* 571.8 → 556.8), for confirmation ^81^Br-DM-TBBPA (C_17_H_16_O_2_Br_2_^81^Br_2_, *m*/*z* 573.8 → 558.8), was monitored. As internal standard, ^13^C-TBBPA (C_3_^13^C_12_H_12_O_2_Br_4_, *m*/*z* 555.8 → 540.8) was used.

As differential matrix effects were observed in the lower calibration range, a matrix-matched calibration was performed for DM-TBBPA analysis in fish by adding the appropriate amount of analyte and internal standard stock solution to 0.5 g of fish matrix. For this, a pooled bream muscle fish matrix was used that was tested to be free of analytes and suitable for method development. The applied method uses 0.5 g as starting material and ends up with a final injectable solution of 1 mL, which corresponds to a constant factor of 2 and yields the respective limit of detection (LOD) and LOQ values as given in Table 1.

**Table 1:** Analytical parameters for the determination of TBBPA, MM- and DM-TBBPA by means of wet weight and dry weight, respectively

Analyte	Matrix	Relative recovery (%)^a^	LOQ (μg kg^−1^ ww/dw)^b^	LOD (μg kg^−1^ ww/dw)^c^
TBBPA	Fish	112.0	0.45	0.1
	SPM	112.0	0.45	0.1
	Sediment	107.8	0.45	0.1
MM-TBBPA	Fish	81.2	0.8	0.2
	SPM	97.7	0.8	0.2
	Sediment	98.6	0.8	0.2
DM-TBBPA	Fish	96.8^b^	1.6	0.4
	SPM	89.1	0.7	0.2
	Sediment	107.7	0.7	0.2

^a^The recovery of the fortification experiment is correlated to the slope of recovery functions as recovery = slope × 100

^b^By means of fortified matrix samples at 4 and 16 μg kg^−1^ ww based on a matrix calibration

^c^The limit of quantification (LOQ) and the limit of detection (LOD) were calculated according to DIN 32645 using the software SQS 2010. Concentrations for fish are given on a basis of wet weights (ww); those for SPM and sediment are given on the basis of dry weights (dw)

The resulting analyte concentration levels ranged from 1 to 20 μg kg^−1^ ww, whereas a basic calibration was used for the determination of DM-TBBPA in SPM and sediment samples with a calibration range from 0.1 to 1 μg kg^−1^ ww. Each calibration consisted of 10 calibration points and contained 10 μg kg^−1^ IS (^13^C-TBBPA). Fortified matrix as quality control samples, as well as blank samples, was part of any sample series to assure continuous analytical quality.

Procedural blanks were part of every measurement series. If appropriate, averaged blank levels were subtracted from sample results accordingly.

For result handling and time trend analysis, all results <LOQ were treated as 0 (zero).

## Results and discussion

### Method development and validation

It was aspired to develop an LC-based multi-method for the simultaneous determination of TBBPA, MM- and DM-TBBPA, not requiring any derivatization in contrast to GC-based methods. However, it turned out that DM-TBBPA could not be ionized with standard ionization techniques, such as ESI^+/−^, in contrast to TBBPA and MM-TBBPA where ESI^−^ is very sensitive. Here, for the first time, we describe a method for the sensitive determination of DM-TBBPA by LC using APPI. Finally, it was possible to develop a simultaneous sample preparation starting with 0.5 g sample material, only requiring a differential measurement of the final extracts. For TBBPA and MM-TBBPA, a measurement by Orbitrap-based LC-HR-MS/MS was performed allowing for lower analytical limits, whereas for DM-TBBPA, a measurement by triple quadrupole MS/MS was applied.

The resulting basic calibration lines for TBBPA and MM-TBBPA obtained by linear regression analysis (0.1–10 μg L^−1^) were used to infer the coefficients of determination as *r* ^2^ = 0.996 and *r* ^2^ = 0.982, respectively. A basic calibration for DM-TBBPA (0.1–10 μg L^−1^) showed a coefficient of determination of *r* ^2^ = 0.982 and a matrix-matched calibration for DM-TBBPA (0.2–20 μg kg^−1^ ww) in bream muscle resulted in *r* ^2^ = 0.989. With a dynamic range of two orders of magnitude and *r* ^2^ of >0.98, the calibrations can be accepted for quantification.

The LOQ and LOD values were derived from the calibration lines by applying a validation suggested by Geiss and Einax [29] using Microsoft Excel AddIn SQS2013. These calibration functions were recorded in solvent/buffer systems, and thus, the inferred parameters are initially given in microgrammes per litre. However, the results are given in microgrammes per kilogramme, since the samples are solid material.

Recovery rates were determined for all analytes in all matrices by analysis of fortified matrix samples and are inferred from the slope of the recovery functions, except for DM-TBBPA in fish muscle where the recovery was experimentally determined by repeated analysis of 10 samples fortified at 4 and 16 μg kg^−1^ ww, each, on the basis of a matrix calibration. The handling and measurement of the fortified samples were identical to the treatment of the test samples. Results of recovery experiments are provided in Table 1. The recoveries for all analytes in all matrices range from 81.2 to 112.0% when derived from regression lines. The precision and accuracy are well in the range required by EU guidance document for pesticide residue analytical methods in environmental matrices, i.e. recovery rates of 70–120% and precision of ≤20% relative standard deviation (RSD) [30]. This is also proven by fortification experiments (*n* = 4) as indicated in Table 2, where the recoveries range from 70.2 to 113.8% and the standard deviation from 3.9 to 15.1% for all analytes in all matrices.

**Table 2:** Analytical parameters for the determination of TBBPA, MM-TBBPA and DM-TBBPA from fortification experiments in bream muscle, SPM and sediment (wet weight data for bream, dry weight data for SPM/sediment)

Matrix	Fortification level (μg kg^−1)^	Parameter	TBBPA	MM-TBBPA	DM-TBBPA
Bream muscle	4	Mean (μg kg^−1^)	4.40	4.30	3.90
		Recovery (%)	110.6	107.1	96.7
		SD (μg kg^−1^)	0.31	0.33	0.53
		RSD (%)	7.0	7.7	13.6
	16	Mean (μg kg^−1^)	15.3	16.9	15.5
		Recovery (%)	95.7	105.3	96.9
		SD (μg kg^−1^)	1.06	1.51	0.87
		RSD (%)	6.9	9.0	5.6
SPM	4	Mean (μg kg^−1^)	3.6	4.55	3.9
		Recovery (%)	90.6	113.8	98.4
		SD (μg kg^−1^)	0.43	0.34	0.42
		RSD (%)	12.0	7.5	10.7
	16	Mean (μg kg^−1^)	14.25	14.9	11.20
		Recovery (%)	89.3	92.8	70.2
		SD (μg kg^−1^)	0.93	1.39	0.43
		RSD (%)	6.5	9.4	3.9
Sediment	4	Mean (μg kg^−1^)	3.50	4.50	4.25
		Recovery (%)	86.9	112.5	105.7
		SD (μg kg^−1^)	0.53	0.42	0.41
		RSD (%)	15.1	9.3	9.7
	16	Mean (μg kg^−1^)	14.50	15.0	11.45
		Recovery (%)	90.4	93.8	71.5
		SD (μg kg^−1^)	0.89	1.83	1.34
		RSD (%)	6.1	12.2	11.7

*n* = 4 *SD* standard deviation, *RSD* relative standard deviation

All fish results were subsequently transformed to the concentration by lipid weight; the lipid contents were taken from Nguetseng et al. as all details on the fish samples were described there as well [26].

A separate fortification experiment was performed with bream muscle matrix at 4 and 16 μg kg^−1^ ww in quadruplicates for all three analytes. The accuracy for all substances at both concentrations was within 95.7 and 111% recovery of nominal values and the precision ranged from 1.8 to 13.6% by means of the RSD. This proves the quality of the method and the applicability in the measurement range. Details of this experiment are given in Table 2. To secure the analytical quality during the measurement of the fish samples, quality control (QC) samples fortified at 4 and 16 μg kg^−1^ were measured as part of all analytical series (at least every 20 injections). During the analysis of fish samples, QC samples were found to have recoveries of 80–120% of nominal. Also, blank samples were part of the analytical series. In the case of fish samples, blank samples showed a background in the level of the LOQ. The actual results were corrected for these background levels and reported.

Mass spectra, example chromatograms, and further details are available as Electronic Supplementary Material (ESM) to this publication.

### Results of sample analysis

The developed methods were applied to a series of samples taken from sampling sites across Europe. In total, 59 samples were analysed in duplicate from five estuary sites and Lake Belau in northern Germany, a freshwater lake as expected unpolluted reference site [31]. From all estuary sites, bream muscle and SPM were analysed. From the Scheldt sampling site in The Netherlands, in addition to bream, sole muscle was analysed. From the freshwater site Lake Belau, surface sediment layers were sampled instead of SPM. The results of all fish sample analyses are comprised in Table 3 and are limited to results, where means of duplicate determinations are above the respective LOQ. Results below the LOQ are listed in brackets and results below LOD are given as symbols (<LOD) only. Results for SPM and surface sediment layers are presented in Table 4.

**Table 3:** Results of the analysis of fish samples (bream and sole) for TBBPA, MM-TBBPA and DM-TBBPA, as well as the fat contents of the fish and the results calculated by means of lipid weight. Individual LOD and LOQ values are given in Table 1

Sample material	Sampling site	Sampling year	TBBPA	MM-TBBPA	DM-TBBPA	Fat content	TBBPA	MM-TBBPA	DM-TBBPA
			Concentration in μg kg^−1^ ww			%	Concentration in μg kg^−1^ lw		
Bream	Western Scheldt	2007	(0.44)	<LOD	<LOD	3.4	(12.9)	<LOD	<LOD
		2008	(0.45)	<LOD	<LOD	2.7	(16.7)	<LOD	<LOD
		2009	(0.40)	<LOD	<LOD	3.0	(13.3)	<LOD	<LOD
		2010	(0.40)	<LOD	<LOD	2.4	(16.7)	<LOD	<LOD
		2011	(0.36)	<LOD	<LOD	5.0	(7.2)	<LOD	<LOD
		2012	(0.36)	<LOD	<LOD	3.9	(9.2)	<LOD	<LOD
		2013	(0.41)	<LOD	<LOD	3.3	(12.4)	<LOD	<LOD
	River Mersey	2007	**0.98**	<LOD	<LOD	2.5	39.2	<LOD	<LOD
		2008	**0.74**	<LOD	<LOD	2.9	25.5	<LOD	<LOD
		2012	**1.19**	**1.63**	<LOD	1.9	62.6	85.8	<LOD
		2013	(0.22)	<LOD	<LOD	2.2	(10.0)	<LOD	<LOD
	River Götaälv	2007	(0.33)	<LOD	<LOD	2.5	(13.2)	<LOD	<LOD
		2008	<LOD	<LOD	<LOD	3.6	<LOD	<LOD	<LOD
		2012	(0.31)	(0.37)	<LOD	3.0	(10.3)	(12.3)	<LOD
		2013	(0.26)	<LOD	<LOD	3.4	(7.6)	<LOD	<LOD
	River Rhone	2007	(0.45)	(0.05)	<LOD	0.8	(60.0)	(6.7)	<LOD
		2008	(0.44)	<LOD	<LOD	2.9	(15.2)	<LOD	<LOD
		2009	**0.65**	(0.61)	<LOD	3.6	18.1	(16.9)	<LOD
		2010	(0.24)	<LOD	<LOD	2.4	(10.0)	<LOD	<LOD
		2011	**0.76**	(0.38)	<LOD	2.1	36.2	(18.1)	<LOD
		2012	**0.49**	<LOD	<LOD	2.5	19.6	<LOD	<LOD
		2013	**0.57**	(0.21)	<LOD	1.7	33.5	(12.4)	<LOD
	River Tees	2007	**0.70**	**1.14**	<LOD	3.3	21.2	34.5	<LOD
		2008	(0.42)	**1.16**	<LOD	3.4	(12.4)	34.1	<LOD
		2009	**0.48**	**1.16**	<LOD	2.7	17.8	43.0	<LOD
		2010	**1.02**	**1.22**	<LOD	1.9	53.7	64.2	<LOD
		2011	**0.91**	**1.20**	<LOD	2.2	41.4	54.5	<LOD
		2012	**0.83**	**1.83**	<LOD	2.4	34.6	76.3	<LOD
		2013	(0.31)	**0.83**	<LOD	2.1	(14.8)	39.5	<LOD
	Lake Belau	2007	(0.36)	**1.40**	<LOD	0.9	(39.1)	152.2	<LOD
		2008	**0.45**	**1.43**	<LOD	2.2	(20.5)	65.0	<LOD
		2009	(0.28)	**0.99**	<LOD	3.1	(9.0)	31.9	<LOD
		2010	(0.32)	(0.51)	<LOD	1.6	(20.0)	(31.9)	<LOD
		2011	(0.22)	(0.44)	<LOD	1.3	(16.9)	(33.8)	<LOD
		2012	(0.26)	(0.24)	<LOD	1.1	(23.6)	(21.8)	<LOD
		2013	(0.23)	(0.35)	<LOD	1.3	(17.7)	(26.9)	<LOD
Sole	Western Scheldt	2007	**0.64**	(0.72)	<LOD	1.0	64.0	(72.0)	<LOD
		2008	**0.73**	**0.92**	<LOD	1.2	60.8	76.7	<LOD
		2009	**0.47**	(0.59)	<LOD	0.9	52.2	(65.6)	<LOD
		2010	**0.48**	**0.84**	<LOD	1.5	32.0	56.0	<LOD
		2011	**0.53**	**1.00**	<LOD	2.3	23.0	43.5	<LOD
		2012	**0.56**	**0.86**	<LOD	1.0	56.0	86.0	<LOD
		2013	**0.68**	(0.62)	<LOD	1.0	68.0	(62.0)	<LOD

Results above LOQ are bold. Results below the LOQ are listed in brackets and results below limit of detection (LOD) are given as symbols (<LOD) only

**Table 4:** Results of the analysis of SPM and surface sediment layer samples for TBBPA, MM-TBBPA and DM-TBBPA. As a characterization parameter regarding binding properties to lipophilic compounds, the total organic carbon (TOC) content is given. Individual LOD and LOQ values are given in Table 1

Sample material	Sampling site	Sampling year	Total organic carbon (TOC)	TBBPA	MM-TBBPA	DM-TBBPA
			%	Concentration in μg kg^−1^ dw		
SPM	River Mersey	2008	**8.2**	**9.44**	**4.48**	(0.65)
	River Göta älv	2008	**3.0**	**1.99**	**2.32**	<LOD
	River Rhone	2008	**5.1**	**1.83**	**2.59**	<LOD
		2010	**1.9**	**2.07**	**3.08**	<LOD
		2012	**2.1**	**1.84**	**2.91**	<LOD
		2014	**2.1**	**3.93**	**3.85**	<LOD
	River Tees	2008	**8.7**	**3.97**	**3.36**	<LOD
		2010	**7.3**	**3.80**	**3.02**	<LOD
		2012	**7.2**	**2.28**	**2.85**	(0.22)
		2014	**6.5**	**3.52**	**3.28**	(0.55)
	Western Scheldt	2008	**2.6**	<LOD	<LOD	<LOD
		2010	**3.0**	**0.53**	<LOD	<LOD
		2012	**3.2**	(0.39)	<LOD	<LOD
		2014	**2.2**	(0.24)	<LOD	<LOD
Surface sediment layer	Lake Belau	2012	**11.1**	**2.58**	**5.51**	<LOD
		2014	**9.7**	**2.30**	**5.16**	<LOD

Results above LOQ are bold. Results below the LOQ are listed in brackets and results below limit of detection (LOD) are given as symbols (<LOD) only

In this study, we found TBBPA in bream muscle from the rivers Rhone, Tees and Mersey for most of the sampling years, but not, or only by occasion, in samples from the Scheldt, Göta älv and Lake Belau. The highest analyte levels were observed in British river estuaries with values of 1.19 μg kg^−1^ ww (62.6 μg kg^−1^ lipid weight (lw)) in 2012 and 1.02 μg kg^−1^ ww (53.7 μg kg^−1^ lw) in 2010 in bream samples from the rivers Mersey and Tees, respectively. For hexabromocyclododecane (HBCD) which was quantified in the same set of samples, also Tees and Mersey revealed the highest levels [25]. While the higher HBCD fish concentrations at the Tees River may be explained by an upstream former HBCD production plant, the UK environment may be exposed generally to higher BFR levels due to stricter fire protection regulations in UK as compared to other European countries [32]. TBBPA is not produced in Europe [12].

Quantifiable concentrations, albeit at low and constant (ww) levels (23–68 μg kg^−1^ lw; 0.47–0.73 μg kg^−1^ ww), were found in sole samples from the Scheldt, in contrast to bream samples from the same site with levels all below LOQ. Levels for TBBPA from the same sampling sites for fish as well as sediment are in line with earlier findings reported by Morris et al. [33].

In fish samples, only where TBBPA was found, also MM-TBBPA was observed at low levels comparable to the concentrations of its parent TBBPA. DM-TBBPA, however, was not detectable in fish matrix at any sampling site.

There was a significant positive correlation (*r* = 0.833; *p* = 0.02, as defined by Pearson product-moment correlation analysis) between the occurrence of TBBPA and MM-TBBPA in fish only at the Lake Belau sampling site. No significant correlation was found for any stream water system. This may be explained by the static system of a lake, where equilibrium between the parent and metabolite may have been established, whereas in streams, the tissue and SPM levels of TBBPA and metabolites may rather reflect a short-term steady-state level of the stream concentration, being influenced, for example, by metabolism in fish, other biota or weather events.

Comparable concentrations of TBBPA and MM-TBBPA were detected in SPM samples from all test sites except the Western Scheldt. Also for SPM, the British rivers Mersey and Tees represent sampling sites with the highest findings at 9.44 and 3.97 μg kg^−1^ dry weight (dw), both in 2008, respectively.

Similar are the findings for MM-TBBPA. In SPM samples from stream sources, the ratio of TBBPA and MM-TBBPA is fluctuating, but at comparable levels, except for the River Mersey (2008), where the MM-TBBPA level is less than half (4.48 μg kg^−1^ dw) of TBBPA (9.44 μg kg^−1^ dw). However, there is a significant positive correlation (*r* = 0.884; *p* = 0.0007, as defined by Pearson product-moment correlation analysis) between the occurrence of TBBPA and MM-TBBPA across all SPM sampling sites and years. As in fish samples, no DM-TBBPA was detected above LOQ.

However, higher levels of TBBPA and derivatives in SPM from the rivers Tees and Mersey and in Lake Belau sediment may also be caused by higher contents of total organic carbon (TOC; Table 4) in these samples. TOC is a fraction often related to the binding of lipophilic compounds. If SPM/sediment data are normalized to TOC levels, the highest levels of TBBPA are found in Rhone SPM in 2014 and Mersey SPM from 2008. TOC-normalized MM-TBBPA is also high in Rhone SPM. Concentrations of both compounds are increasing: TBBPA from 36 to 187 μg kg^−1^ TOC dw and MM-TBBPA from 51 to 183 μg kg^−1^ TOC dw in the period 2008–2014. The TOC-normalized data are available as ESM to this publication.

At the Scheldt, only for the sampling year 2010, low levels of TBBPA (0.53 μg kg^−1^ dw) were found in respective SPM, which may have caused a relatively higher exposure for soles living in sediment compared to bream. This may explain why TBBPA was found in sole, but not in bream. Bream and sole share their feed (worms, molluscs and small crustaceans in the sediment or at the sediment/water interface), but soles live in closer contact to the sediment. Moreover, bream in the Scheldt are caught near the river banks while the soles are caught also in the midstream area where the sediment is more influenced by re-mobilization and deposition. Thus, sediment and feed organisms from different sites of the stream bed may cause different TBBPA levels, eventually leading to a different TBBPA exposure of the fish. Similar observations were made for the BFR HBCD (unpublished own work; manuscript submitted).

Also, in surface sediment layer samples from Lake Belau, TBBPA was found at concentrations above 2 μg kg^−1^ dw which is in the mid-range, compared to findings in SPM from European river estuaries. In surface sediment layers, in contrast to SPM, MM-TBBPA levels are not lower or similar, but more than twice as high as TBBPA with levels above 5 μg kg^−1^ dw.

The findings for TBBPA are in line with samples from the year 2004, where sediments from the Western Scheldt are comparably low (0.3–1.3 μg kg^−1^ on a dw basis) and higher in samples from British rivers (ranging from <2.4 to 57 μg kg^−1^ dw, with a peak at 9750 μg kg^−1^ dw in sediments from the River Skerne in England) [33]. In a study by Cunha et al. which analysed TBBPA in marine fish, no TBBPA could be found [22].

Interestingly, no DM-TBBPA was found in any of the fish, SPM or surface sediment layer samples from all sites, albeit it is considered the endpoint of environmental TBBPA by means of microbial O-methylation [17]. However, corresponding to our findings, Peng et al. [34] only found MM-TBBPA as a result of freshwater algae metabolism along with some other metabolites, but no DM-TBBPA. The literature data for DM-TBBPA are very scarce, especially for environmental monitoring data. A study by Sellstrom and Jansson [35] found DM-TBBPA levels of 24 μg kg^−1^ in river sediment upstream a plastic factory in Sweden, an influence of the respective industry can thus be excluded and other sources must account. A further study by Vorkamp et al. [19], however, found DM-TBBPA levels in peregrine falcon egg samples, sampled from 1986 to 2003, at levels up to 940 μg kg^−1^ lw. DM-TBBPA was found in 29 out of 33 samples. In the same study, no TBBPA was found above LOQ.

A retrospective environmental monitoring approach longs to identify temporal trends of the analytes. However, no time-dependent correlation could be identified for TBBPA, MM-TBBPA or DM-TBBPA. This is on the one hand based on the fact that most of the samples were below the LOQ and on the other hand on the limited sample material available for this study. For samples above LOQ, no clear time trend could be observed. The levels for TBBPA and MM-TBBPA rather scatter around a long-term median, in stream waters, as well as in SPM of stream water systems. The missing of time trends may also indicate a reduced or discontinued emission of TBBPA and its derivatives. Also, Vorkamp et al. [19] could not find a time trend for DM-TBBPA in a retrospective monitoring approach with peregrine falcon eggs dating from 1986 to 2003.

Published methods for the determination of TBBPA, MM-TBBPA and DM-TBBPA indicating method parameters such as LOD or LOQ are quite scarce. Frederiksen et al. [24] developed a differential method for TBBPA and DM-TBBA from fish matrix (liver and blubber) and reached LODs of 550–4200 ng kg^−1^ fish matrix via LC-MS/MS and of 350–2600 ng kg^−1^ fish matrix using GC-NCI-MS, respectively, and is well comparable to our LOD of 100 and 400 ng kg^−1^ fish matrix for TBBPA and DM-TBBPA.

Published methods for TBBPA are more available, and a recent review by Abdallah lists this work comprehensively [10]. The LODs, if given, span from 0.3 ng kg^−1^ to 200 mg kg^−1^ respective matrix, including biological matrices such as fish muscles, human milk, sediment and sewage sludge. Most of the methods published so far use a comparably elaborate sample preparation procedure, involving multiple steps, e.g. extraction of 20 g sample material, gel permeation chromatography (GPC), liquid-liquid extraction (LLE), acidic matrix hydrolysis and subsequent filtration [36]. In latter study, 0.3 ng kg^−1^ ww was achieved as LOD, which is by far the lowest LOD documented for TBBPA. Cunha et al. recently published a QuECHERS-based sample preparation method in combination with LLE where an LOQ of 0.06 μg kg^−1^ ww in fish matrix was reached for TBBPA. This is a promising sample preparation which may also be tried to work as a common preparation for TBBPA and its metabolites. Labadie et al. published a GC-NCI-MS method, for example, yielding an LOD of 50 ng kg^−1^ dw for sediment, with ours being quite similar with 100 ng kg^−1^ dw sediment and SPM [37]. In summary, our method yields competitive detection and quantification limits, albeit applying a comparably simple and straightforward sample processing requiring only 0.5 g starting material.

## Electronic supplementary material

ESM 1 (PDF 1.39 mb)
