# Supplementary material for: Detection of tetrabromobisphenol A and its mono- and dimethyl derivatives in fish, sediment and suspended particulate matter from European freshwaters and estuaries
Source: Anal Bioanal Chem. 2017 Mar 21;409(14):3685–94. doi: 10.1007/s00216-017-0312-z (PMC5406432; doi:10.1007/s00216-017-0312-z)
Supplement: Supplementary file 1 — (PDF 1.39 mb) [file 216_2017_312_MOESM1_ESM.pdf]

Analytical and Bioanalytical Chemistry

Electronic Supplementary Material

Detection of tetrabromobisphenol A and its mono- and dimethyl derivatives in fish, sediment and suspended particulate matter from European freshwaters and estuaries

Matthias Kotthoff, Heinz Rüdel, Heinrich Jürling

1 Results normalized to the TOC

Table S1 is to provide all results for SPM and surface sediment layer normalized on the TOC of the respective sample material.

sample material	sampling site	sampling year	TOC	TBBPA	MM-TBBPA	DM-TBBPA
			%	concentration in µg kg^{-1} dw TOC		
SPM	River Mersey	2008	8.2	**115**	**55**	**8**
	River Göta älv	2008	3.0	**66**	**77**	< LOD
	River Rhone	2008	5.1	**36**	**51**	< LOD
		2010	1.9	**109**	**162**	< LOD
		2012	2.1	**88**	**139**	< LOD
		2014	2.1	**187**	**183**	< LOD
	River Tees	2008	8.7	**46**	**39**	< LOD
		2010	7.3	**52**	**41**	< LOD
		2012	7.2	**32**	**40**	**3**
		2014	6.5	**54**	**50**	**8**
	Western Scheldt	2008	2.6	< LOD	< LOD	< LOD
		2010	3.0	**18**	< LOD	< LOD
		2012	3.2	**12**	< LOD	< LOD
		2014	2.2	**11**	< LOD	< LOD
surface sediment layer	Lake Belau	2012	11.1	**23**	**50**	< LOD
		2014	9.7	**24**	**53**	< LOD

1.1 Analysis of TBBPA and TBBPA-MME by LC-HR-MS

1.1.1 Instrumental Parameters

HPLC-Parameters

HPLC: UPLC Acquity, Waters
Mass spectrometer: Q-Exactive, Thermo Scientic (High resolution system)
Analytical column: 100 x 2.1 mm BEH C18, 1.7 µm, Waters
Flow: 0.35 mL/min
Injection volume: 10 µL
Column temperature: 55 °C
Ionization mode: Electrospray negative (ES-)

Table S2 UPLC Gradient program

Solvent A: water - methanol (95+5, v/v) + 2 mM ammonium acetate
Solvent B: methanol + 2 mM ammonium acetate

Time (min)	A%	B%	flow (mL/min)	curve
0	100	0	0.35	Initial
10	0	100	0.35	6
12	0	100	0.35	1
15	100	0	0.35	1

Table S3 Accurate masses of analytes for high resolution MS

No.	Chemical name	Formula	Accurate mass (M-H)
1	Tetrabromo-bisphenol-A<br>Bromine isotop 81 used for quantitation	$C_{15}H_{12}Br_4O_2$<br>$C_{15}H_{11}O_2Br_2{}^{81}Br_2$	538.74957<br>542.74554
2	Tetrabromo-bisphenol-A, monomethyl ether<br>Bromine isotop 81 used for quantitation	$C_{16}H_{14}Br_4O_2$<br>$C_{16}H_{13}O_2Br_2{}^{81}Br_2$	552.76430<br>556.76030
3	Tetrabromo-bisphenol-A, (ring-$^{13}C_{12}$) (IS)<br>Bromine isotop 81 used for quantitation	$C_3{}^{13}C_{12}H_{12}Br_4O_2$<br>$C_3{}^{13}C_{12}H_{12}O_2\ Br_2{}^{81}Br_2$	550.78896<br>554.78540

1.1.2 Calibration (TBBPA and TBBPA-MME)

Calibration was performed using the calibration solutions no. 1 to 10 in **Fehler! Verweisquelle konnte nicht gefunden werden.** (concentration range 0.1 µg/L to 1.0 µg/L). Each calibration solution contained 10 µg/L internal standard (tetrabromo-bisphenol-A, ring-$^{13}C_{12}$). The resulting calibration lines for TBBPA and TBBPA-MME obtained by linear regression analysis are shown in Fig. **S1**. The parameters of the calibration lines as well as the coefficients of correlation are also shown in Fig. S1.

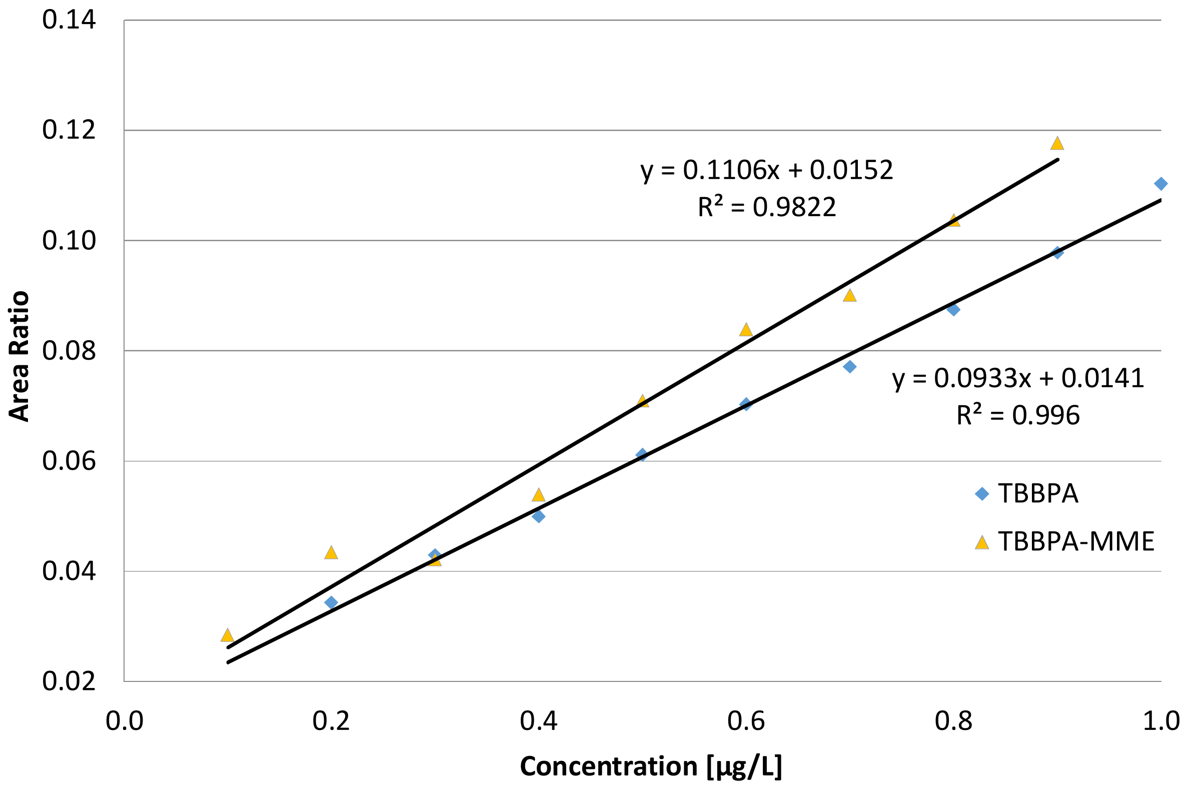

Fig. S1 Calibration lines for TBBPA and TBBPA-MME

1.1.3 Fortification experiments (TBBPA and TBBPA-MME)

The recoveries of the analytes (TBBPA and TBBPA-MME) in the different matrices were determined by fortification experiments (Table S4).

Table S4 Fortification experiments with TBBPA and TBBPA-MME

Matrix	Sample amount [g]	Number fortification levels	Concentration levels [µg/kg ww]
Bream muscle	0.5	10	2, 4, 6, 8, 10, 12, 14, 16, 18, 20
SPM	0.5	10	2, 4, 6, 8, 10, 12, 14, 16, 18, 20
Sediment	0.5	4	6, 10, 14, 18

Each sample was fortified with 100 µL of internal standard stock solution and with 100 µL of a calibration solution according to **Fehler! Verweisquelle konnte nicht gefunden werden.** (calibration solutions no. 10 to 19 for fish and SPM samples, resp. no. 12, 14, 16, 18 for sediment samples). The handling and measurement of the fortified samples was identical to the treatment of the test samples.

The results of the fortification experiments for the different matrices are shown in Fig. S2 to Fig. S4.

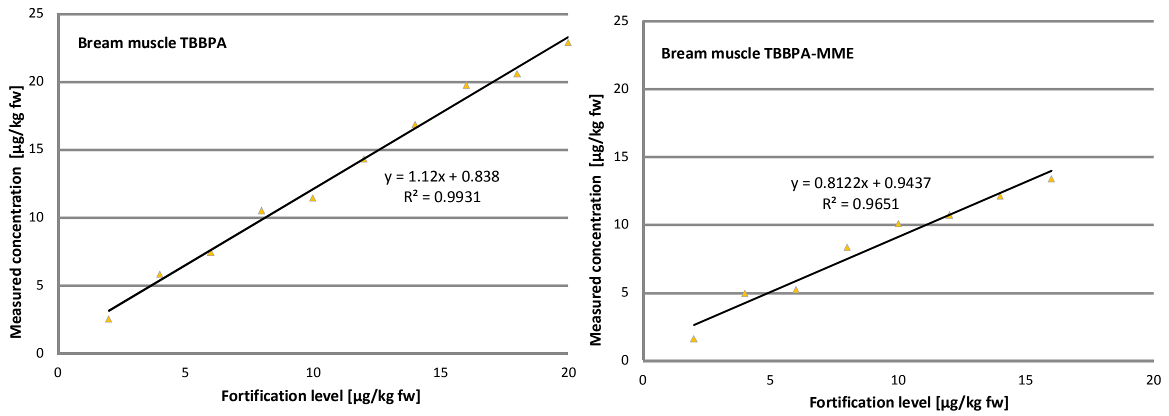

Fig. S2 Recovery functions of TBBPA (left) and TBBPA-MME (right) for bream muscle samples

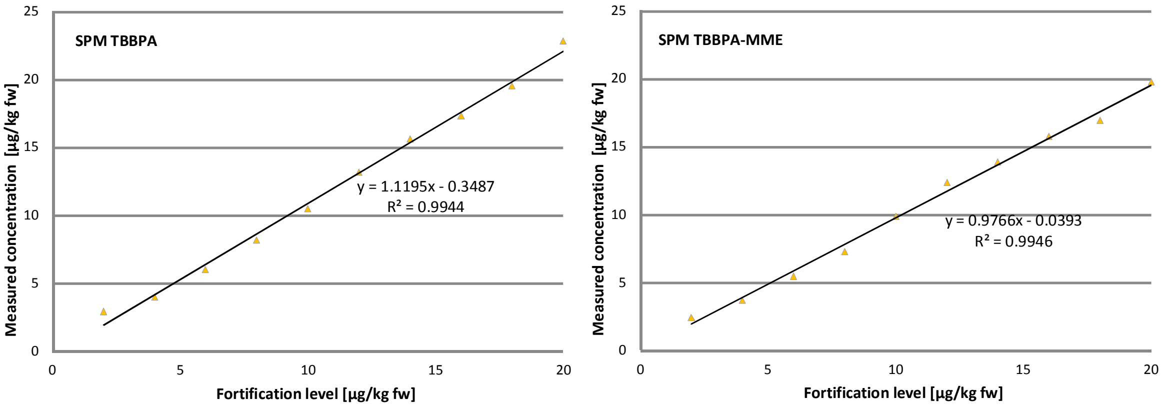

Fig. S3 Recovery functions of TBBPA (left) and TBBPA-MME (right) for SPM samples

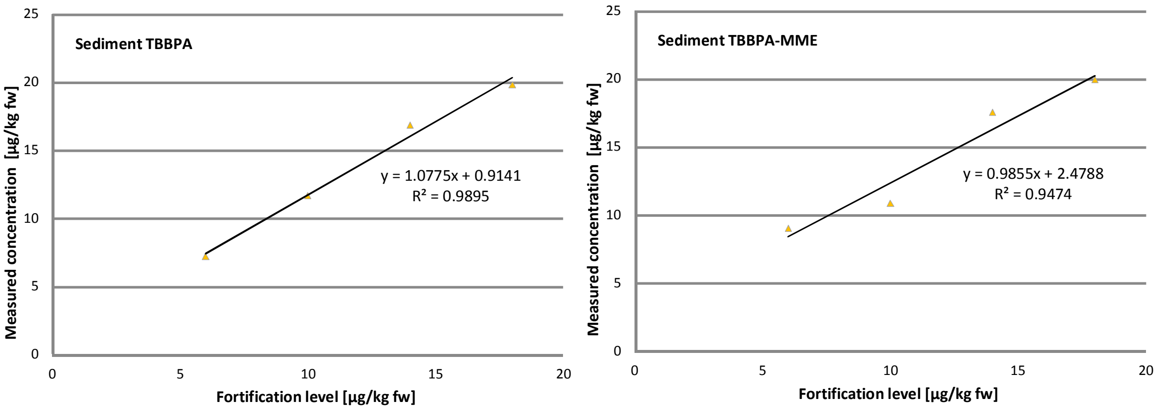

Fig. S4 Recovery functions of TBBPA (left) and TBBPA-MME (right) for sediment samples

The recovery of the fortification experiment is correlated to the slope of the recovery functions as follows: Recovery = Slope *100.

The resulting recoveries of the analytical method are shown in Table S5.

Table S5 Recoveries of TBBPA and TBBPA-MME

Matrix	Analyte	Recovery
Bream muscle	TBBPA	112.0 %
	TBBPA-MME	81.2 %
SPM	TBBPA	112.0 %
	TBBPA-MME	97.7 %
Sediment	TBBPA	107.8 %
	TBBPA-MME	98.6 %

1.2 Analysis of TBBPA-DME by LC-APPI-MS/MS

1.2.1 Instrumental Parameters

HPLC-Parameters

HPLC:	UPLC Acquity, Waters
Mass spectrometer:	TQ-S, Waters (Triple quadrupole system)
Analytical column:	50 x 2.1 mm BEH C18, 1.7 µm, Waters
Flow:	0.05 mL/min
Injection volume:	25 µL
Column temperature:	55 °C
Ionisation mode:	Atmospheric pressure photoionization positive (APPI+)<br>APPI/APCI Dual Source, Waters

Table S6 UPLC Gradient program

Time (min)	A%	B%	flow (mL/min)	curve
0	100	0	0.05	Initial
10	0	100	0.05	6
17	0	100	0.05	1
30	100	0	0.05	1

Solvent A: water - methanol (95+5, v/v) + 2 mM ammonium acetate

Solvent B: methanol + 2 mM ammonium acetate

MS-parameters

Table S7 Masses of analytes; For confirmation purposes a second mass transition (m/z 573.8 -> 558.8) was measured for TBBPA-DME

Chemical name	Formula	Parent Ion for APPI	Fragment Ion for APPI (M-15)
Tetrabromo-bisphenol-A, dimethyl ether Bromine isotop 81 used for quantitation	$C_{17}H_{16}Br_4O_2$<br>$C_{16}H_{14}O_2Br_2{}^{81}Br_2$	Primary transition: 571.8 Confirmatory transition: 573.8	Primary transition: 556.8 Confirmatory transition: 558.8
Tetrabromo-bisphenol-A, (ring-$^{13}C_{12}$) (IS) Bromine isotop 81 used for quantitation	$C_3{}^{13}C_{12}H_{12}Br_4O_2$<br>$C_3{}^{13}C_{12}H_{12}O_2$ $Br_2{}^{81}Br_2$	555.8	540.8

Table S8 The primary mass transitions of TBBPA, TBBPA-MME and TBBPA-DME

Compound	Precursor ion [m/z]	Product ion [m/z]	Dwell time [sec]	Cone voltage [V]	Collision energy [eV]
TBBPA	543.8	528.8	0.15	20	20
TBBPA-MME	557.8	542.8	0.15	20	20
TBBPA-DME	571.8	556.8	0.15	20	20
TBBPA-$^{13}C_{12}$ (IS)	555.8	540.8	0.15	20	20

1.2.2 Calibration (TBBPA-DME)

Calibration for the matrices SPM and sediment was performed using the calibration solutions no. 1 to 6 in **Fehler! Verweisquelle konnte nicht gefunden werden.** (concentration range 0.1 µg/L to 0.6 µg/L). Each calibration solution contained 10 µg/L internal standard (tetrabromo-bisphenol-A, ring-$^{13}C_{12}$). The resulting calibration line for TBBPA-DME obtained by linear regression analysis is shown in Fig. S5. The parameters of the calibration line as well as the coefficients of correlation are also shown in Fig. S5.

This calibration could not be used for the fish muscle samples as the recovery was too low. Therefore matrix calibration was performed for the evaluation of the fish muscle samples.

Ten bream muscle calibration samples were prepared by adding the appropriate amount of analyte and internal standard stock solution to 0.5 g of matrix. The resulting analyte concentration levels were: 2, 4, 6, 8, 10, 12, 14, 16, 18, 20 µg/kg ww. Fig. **S6** shows the matrix calibration line obtained by linear regression analysis.

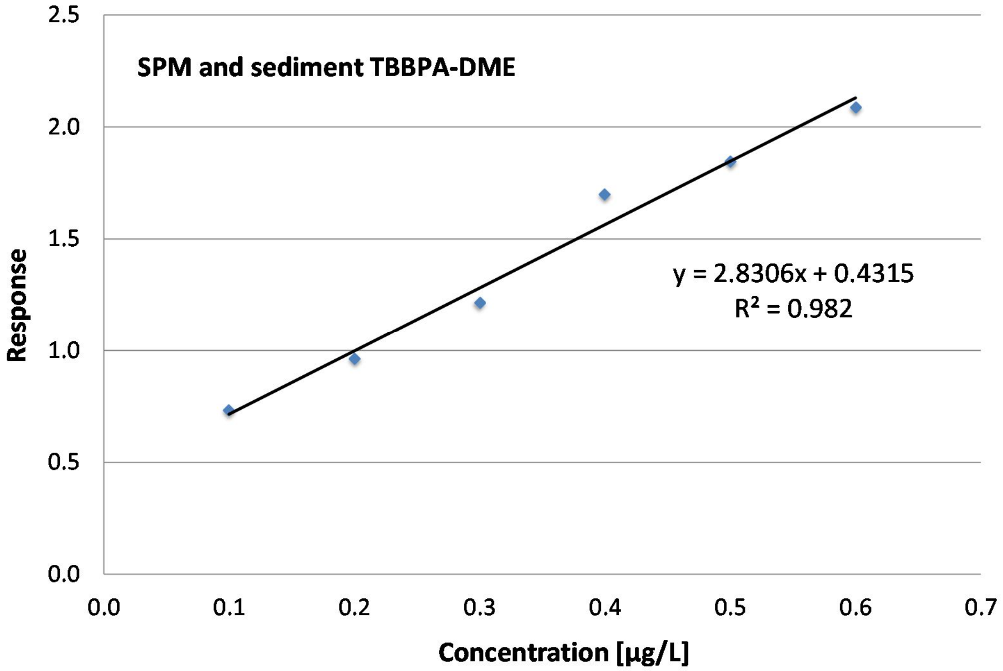

Fig. S5 Calibration line for TBBPA-DME, used for SPM and sediment samples

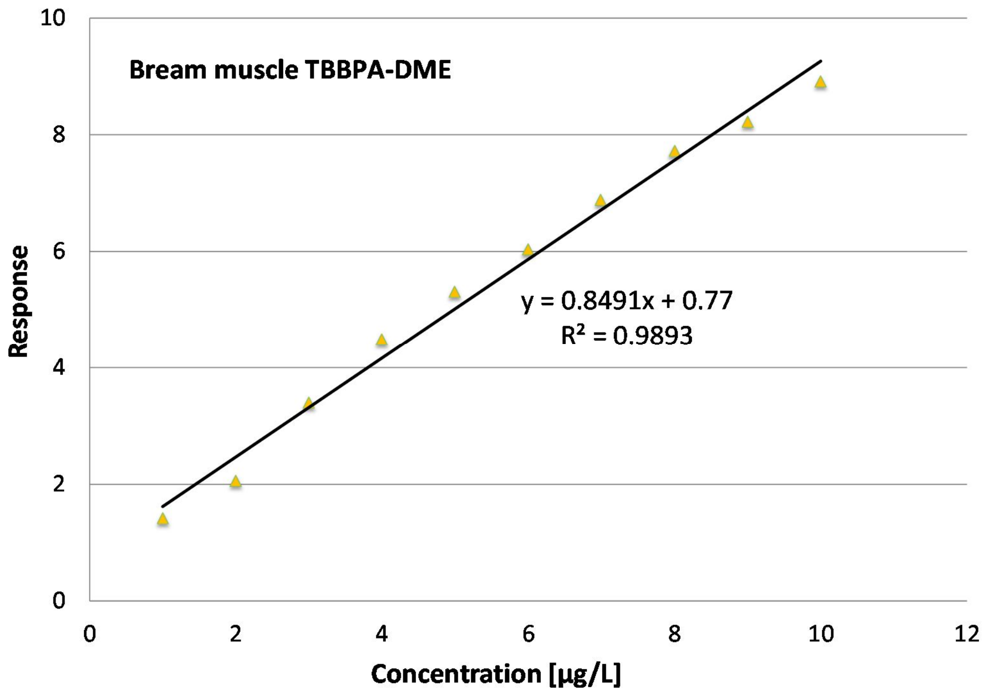

Fig. S6 Matrix calibration line for TBBPA-DME, used for fish muscle samples

1.2.3 Fortification experiments (TBBPA-DME)

The recoveries of the analyte TBBPA-DME) in SPM and sediment matrix were determined by fortification experiments (Table S4). They were not necessary for fish muscle matrix as matrix calibration was applied to this matrix (see chapter 1.2.2).

Table S9 Fortification experiments with TBBPA-DME

**Matrix**	**Sample amount [g]**	**Number fortification levels**	**Concentration levels [µg/kg ww]**
SPM	0.5	10	2, 4, 6, 8, 10, 12, 14, 16, 18, 20
Sediment	0.5	4	2, 6, 10, 14, 18

Each sample was fortified with 100 µL of internal standard stock solution and with 100 µL of a calibration solution according to **Fehler! Verweisquelle konnte nicht gefunden werden.** (calibration solutions no. 10 to 19 for fish and SPM samples, resp. no. 12, 14, 16, 18 for sediment samples). The handling and measurement of the fortified samples was identical to the treatment of the test samples (see chapter 1.3).

The results of the fortification experiments for matrices SPM and sediment are shown in Fig. S7 and Fig. S8.

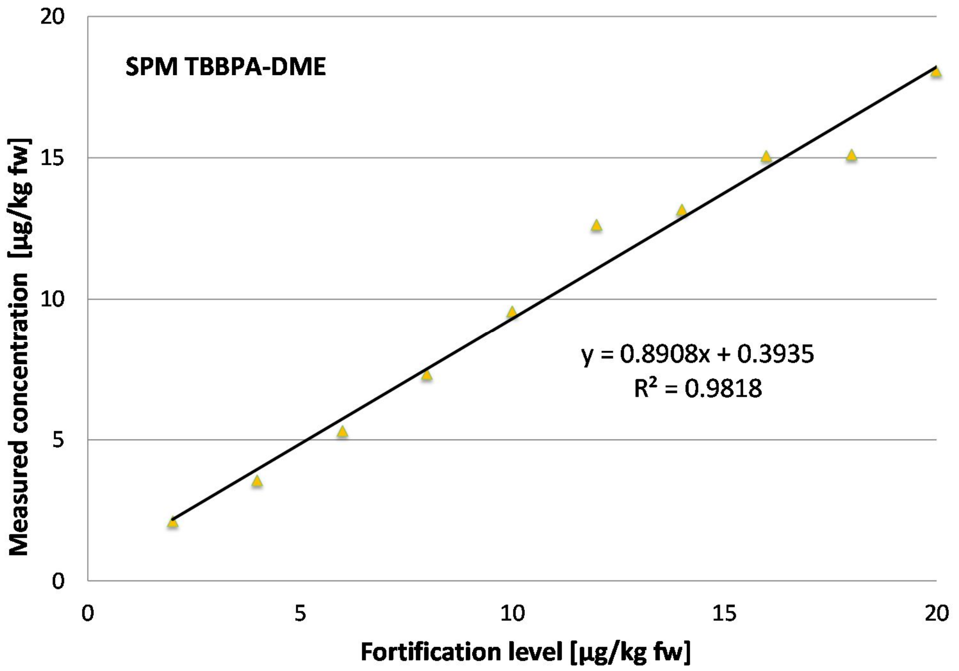

Fig. S7 Recovery function of TBBPA-DME for SPM samples

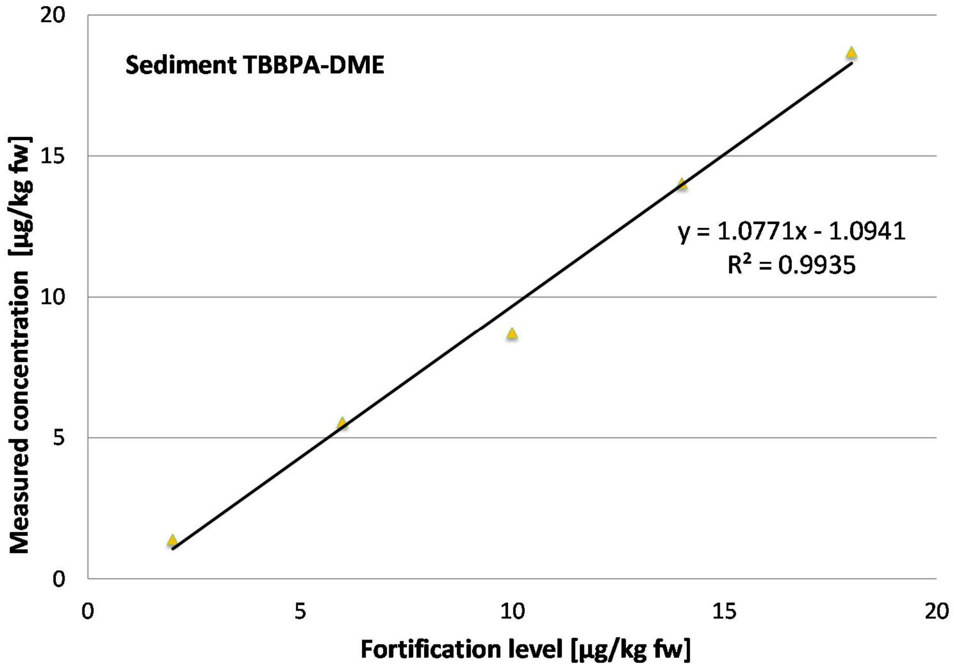

Fig. S8 Recovery function of TBBPA-DME for sediment samples

The recovery of the fortification experiment is correlated to the slope of the recovery functions as follows: Recovery = Slope *100.

The resulting recoveries of the analytical method are shown in Table S5.

Table S10 Recoveries of TBBPA-DME

Matrix	Analyte	Recovery
SPM	TBBPA-DME	89.1 %
Sediment	TBBPA-DME	107.7 %

1.3 Preparation and measurement of samples

Approximately 0.5 g sample (bream muscle or SPM or sediment) is transferred into a 15 mL PP centrifugation tube.

Only for fish muscle samples: 500 µL of concentrated sulfuric acid is added and the samples treated for 10 min in an ultra sonic bath (to degrade lipid materials).

Then 100 µL of the solution of the internal standard (c=100 µg/L) and 5 mL extraction solvent (dichloromethane/n-hexane, 3+1, v/v) are added. The samples are homogenized with an ultra turrax for 1 min and vortexed for 20 min. Then the samples are centrifuged at 4000 rpm for 5 min. The supernatants are transferred into another 15 mL PP centrifugation tube and the extraction procedure is repeated again two times.

The combined 15 mL extraction solvents are removed in a nitrogen evaporator at 40°C to dryness and the residues are resolved in 1 mL acetonitrile-water (50+50, v/v) using an ultrasonic bath for 5 min. Each extract is filtered using 25 mm x 0.45 µm RC filter cartridges directly into 1.5 mL autosampler vials. The samples are now ready for LC-HR-MS resp. LC-APPI-MS/MS analysis.

2 Annex 2: Example chromatograms

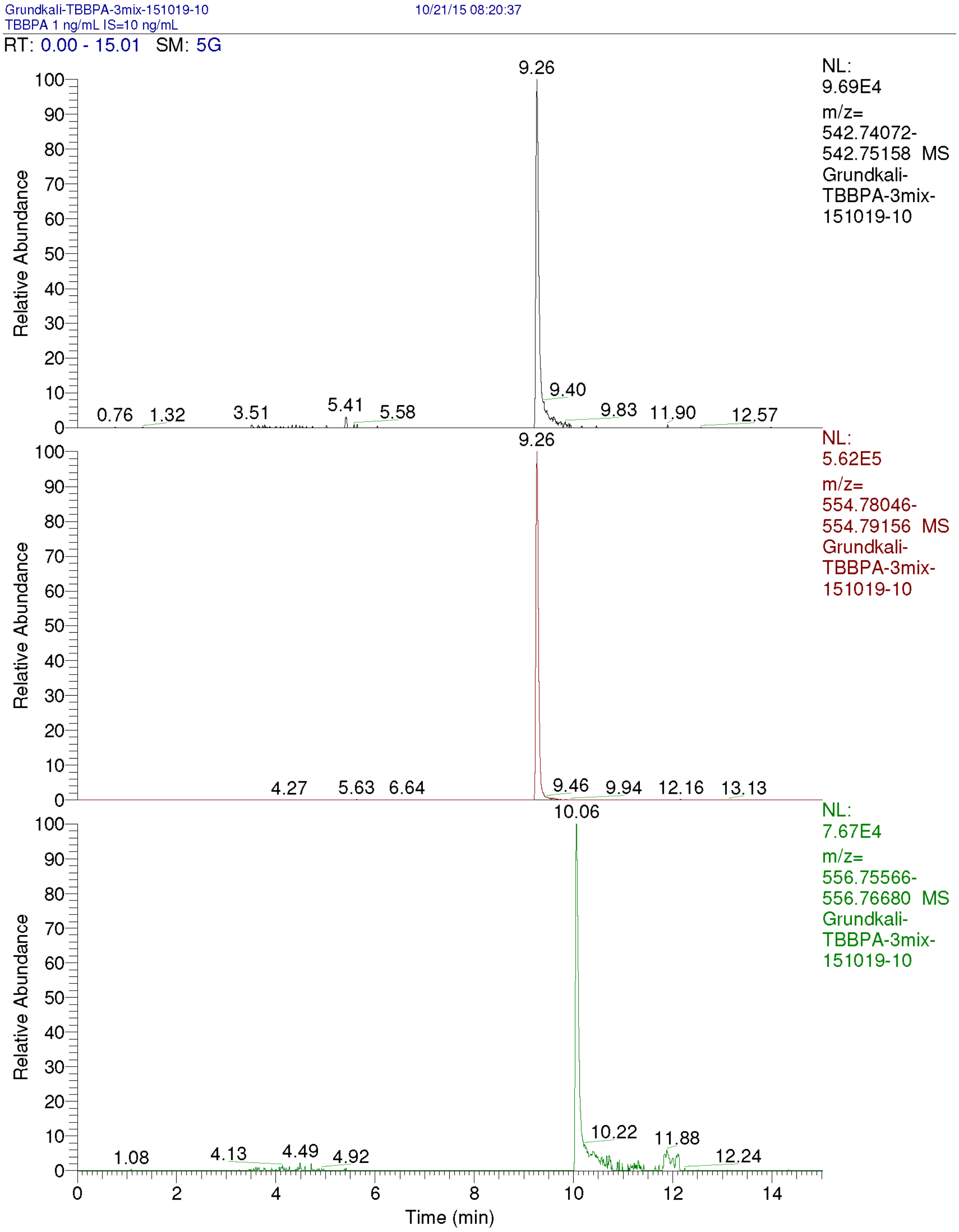

Fig. S9 Calibration sample 1 µg/L: TBBPA (top) and TBBPA-MME (bottom), [middle: internal standard ^{13}C-TBBPA]

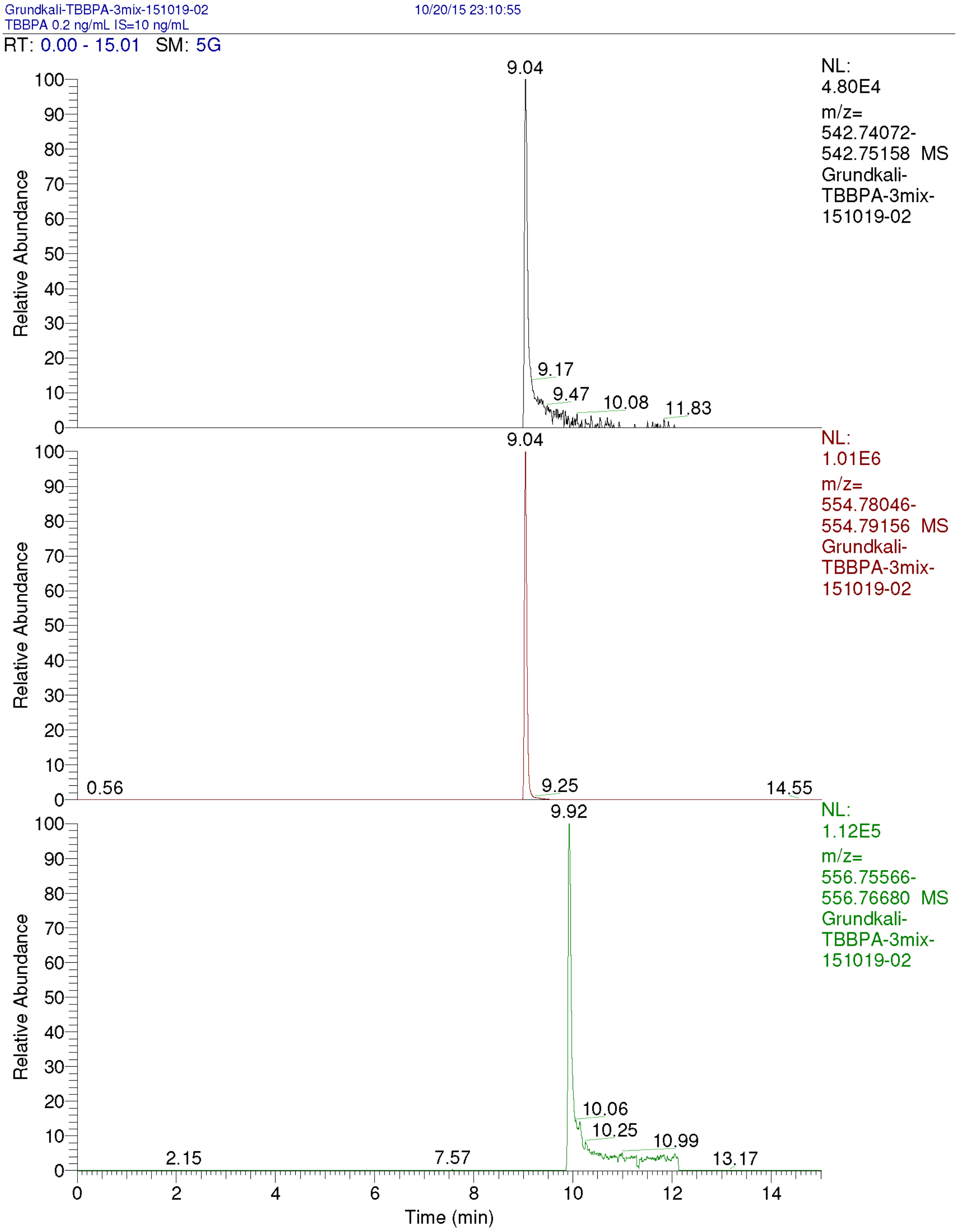

Fig. S10 Calibration sample 0.2 µg/L: TBBPA (top) and TBBPA-MME (bottom), [middle: internal standard ^{13}C-TBBPA]

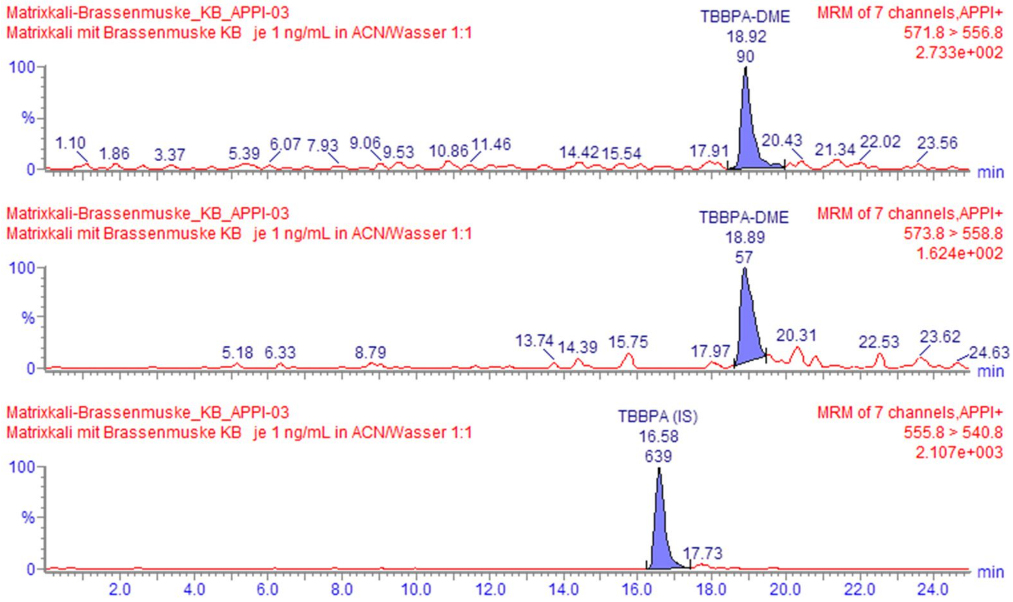

Fig. 11 Matrix-calibration sample bream muscle: 2 µg/kg ww TBBPA-DME

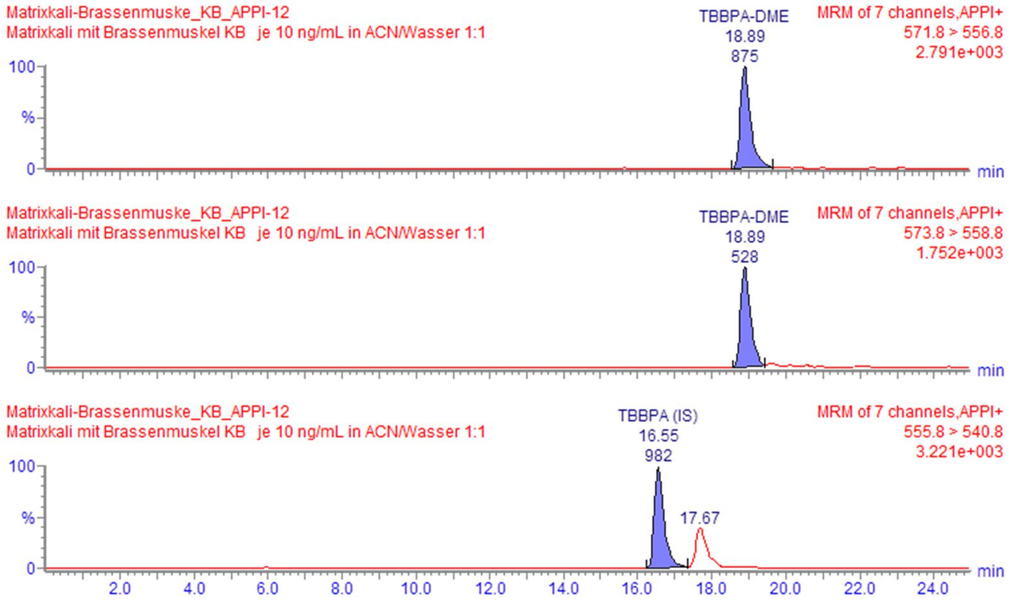

Fig. S12 Matrix-calibration sample bream muscle: 20 µg/kg ww TBBPA-DME

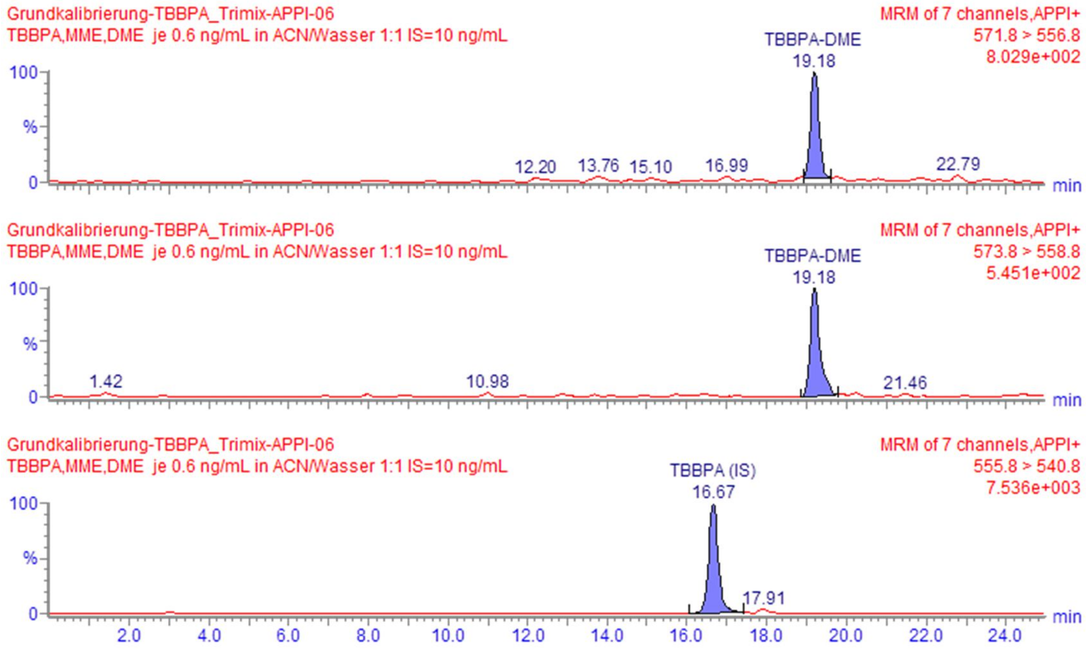

Fig. S13 Basic calibration sample 0.6 ng/mL TBBPA-DME

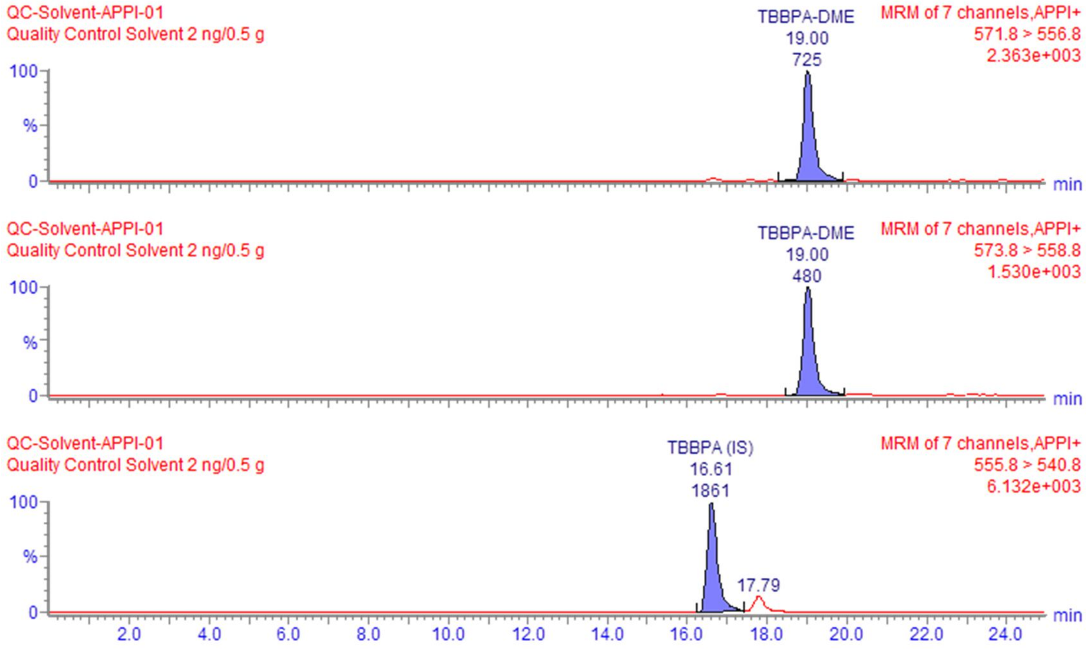

Fig. S14 Quality Control sample 2 µg/L TBBPA-DME

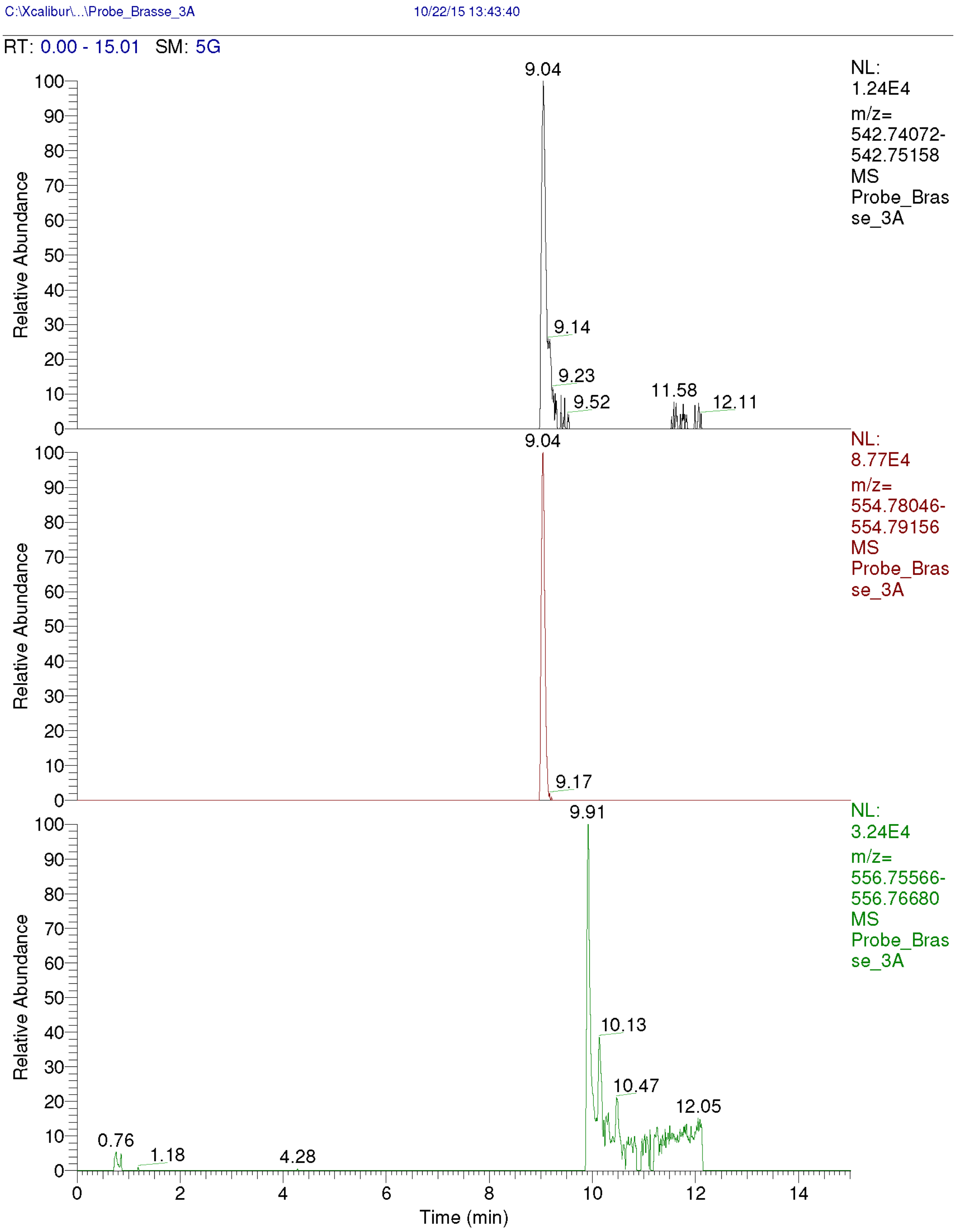

Fig. S15 TBBPA (top) and TBBPA-MME (bottom) in Bream sample River Mersey, Great Britain (Sample 3 A) [middle: internal standard ^{13}C-TBBPA]

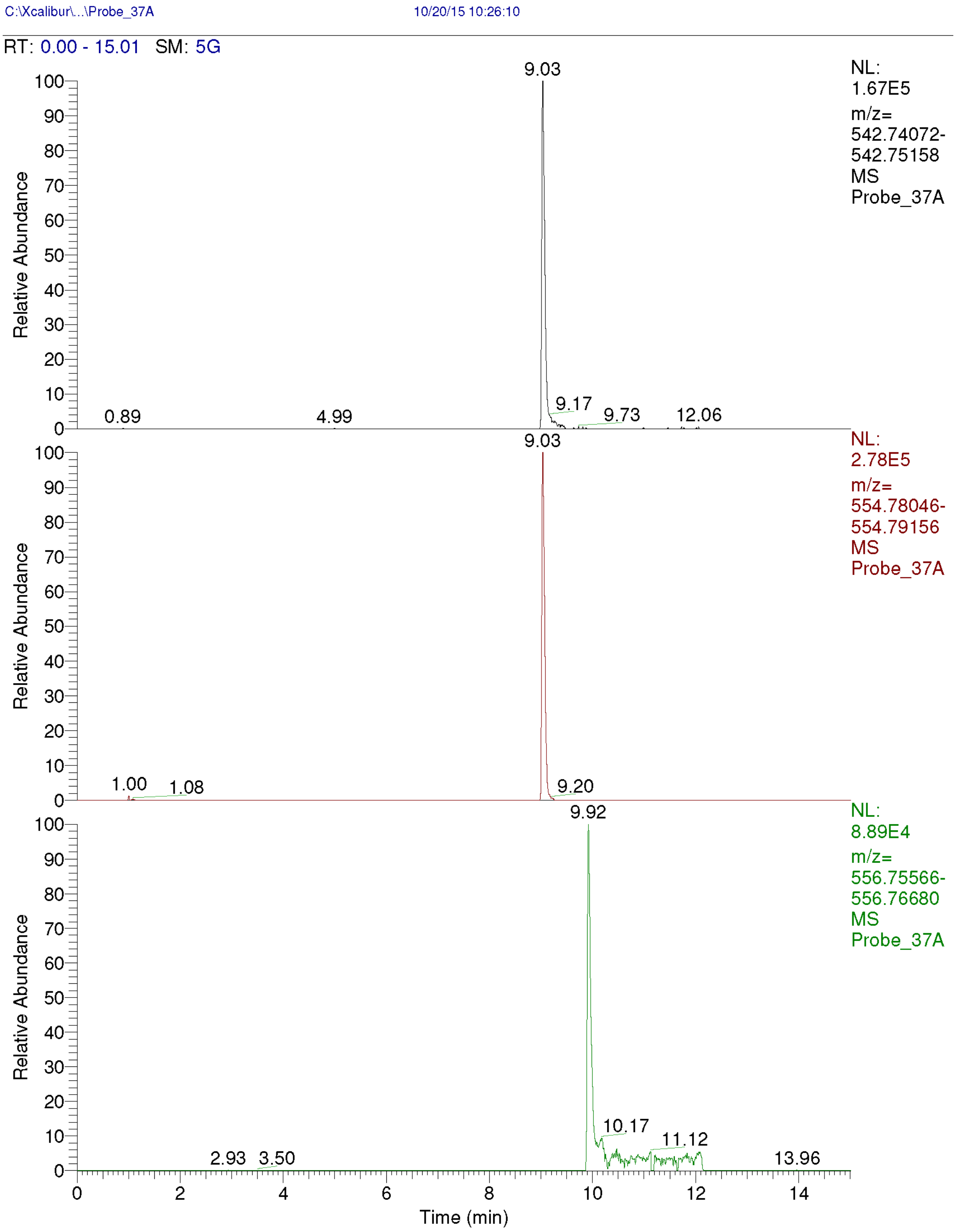

Fig. S16 TBBPA (top) and TBBPA-MME (bottom) in SPM sample River Mersey, Great Britain (Sample 37 A) [middle: internal standard ^{13}C-TBBPA]

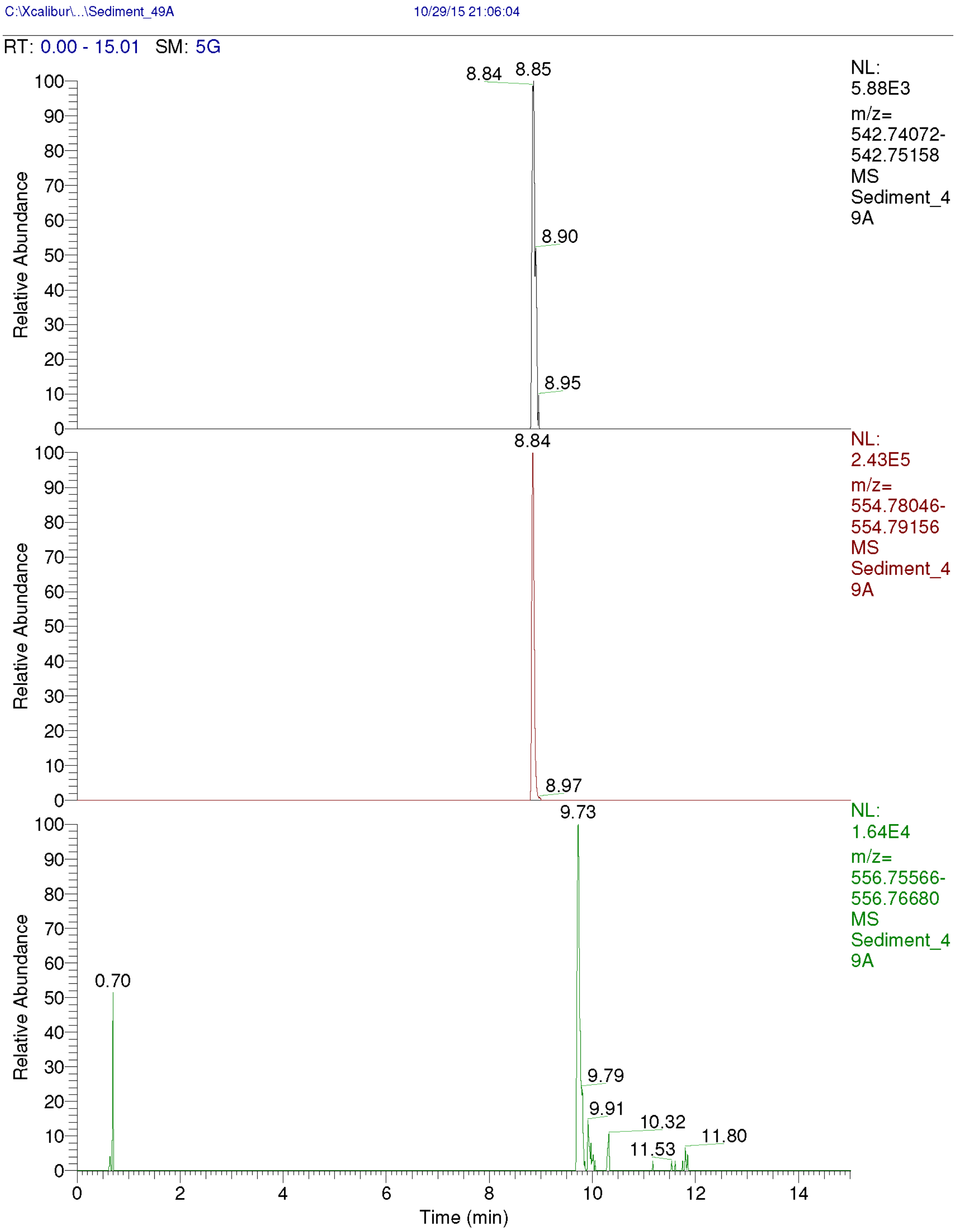

Fig. S17 TBBPA (top) and TBBPA-MME (bottom) in sediment sample Lake Belau, Germany (Sample 49 A) [middle: internal standard ^{13}C-TBBPA]

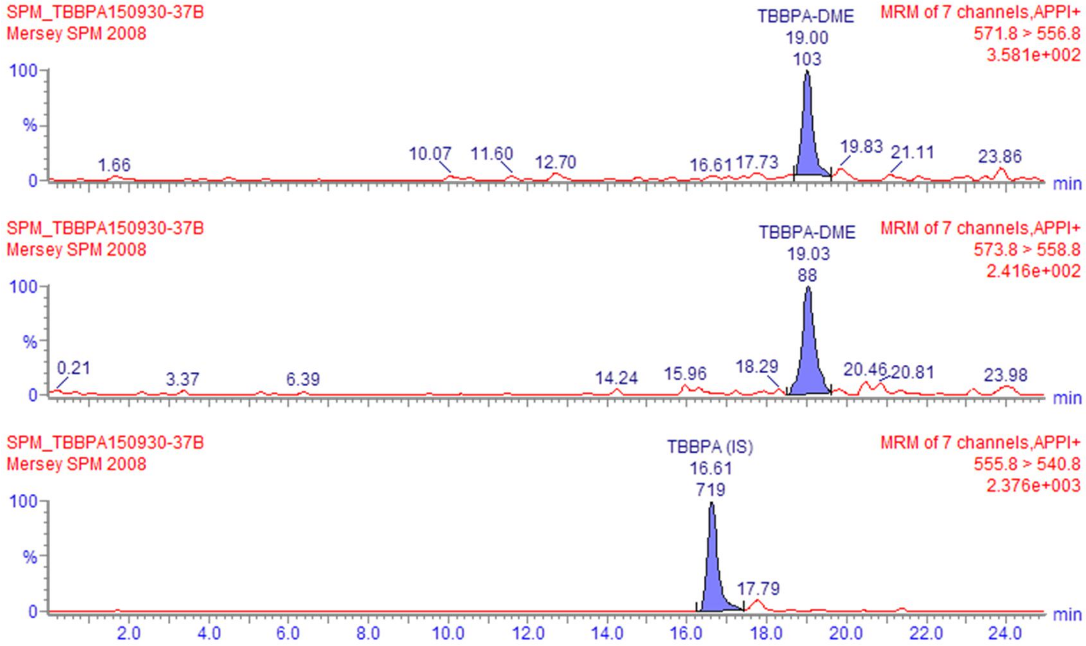

Fig. S18 TBBPA-DME in SPM sample River Mersey, Great Britain (Sample 37 B)
